# Role of the mesenchymal stromal cells in bone marrow failure of Fanconi Anemia patients

**DOI:** 10.3389/fcell.2024.1286815

**Published:** 2024-07-25

**Authors:** Josune Zubicaray, Maria Ivanova, June Iriondo, Jorge García Martínez, Rafael Muñoz-Viana, Lorea Abad, Lorena García-García, Jesús González de Pablo, Eva Gálvez, Elena Sebastián, Manuel Ramírez, Luis Madero, Miguel Ángel Díaz, África González-Murillo, Julián Sevilla

**Affiliations:** ^1^ Hematology and Hemotherapy Unit, Pediatric Onco-hematology Department, Hospital Infantil Universitario Niño Jesús, Madrid, Spain; ^2^ Advanced Therapy Unit, Oncology, Fundación para la Investigación Biomédica Hospital Infantil Universitario Niño Jesús, Madrid, Spain; ^3^ Instituto de Investigación Sanitaria Hospital Universitario de La Princesa (IIS-Princesa), Madrid, Spain; ^4^ Department of Pediatric Hematology and Oncology, Hospital Infantil Universitario Niño Jesús, Madrid, Spain; ^5^ Hematopoietic Stem Cell Transplant Unit, Hospital Infantil Universitario Niño Jesús, Madrid, Spain

**Keywords:** mesenchymal stromal cells, bone marrow microenvironment, bone marrow failure, gene therapy, Fanconi anemia

## Abstract

**Introduction:**

Fanconi anemia (FA) is an inherited disorder characterized by bone marrow failure, congenital malformations, and predisposition to malignancies. Alterations in hematopoietic stem cells (HSC) have been reported, but little is known regarding the bone marrow (BM) stroma. Thus, the characterization of Mesenchymal Stromal Cells (MSC) would help to elucidate their involvement in the BM failure.

**Methods:**

We characterized MSCs of 28 FA patients (FA-MSC) before and after treatment (hematopoietic stem cell transplantation, HSCT; or gene therapy, GT). Phenotypic and functional properties were analyzed and compared with MSCs expanded from 26 healthy donors (HD-MSCs). FA-MSCs were genetically characterized through, mitomycin C-test and chimerism analysis. Furthermore, RNA-seq profiling was used to identify dysregulated metabolic pathways.

**Results:**

Overall, FA-MSC had the same phenotypic and functional characteristics as HD-MSC. Of note, MSC-GT had a lower clonogenic efficiency. These findings were not confirmed in the whole FA patients’ cohort. Transcriptomic profiling identified dysregulation in HSC self-maintenance pathways in FA-MSC (HOX), and was confirmed by real-time quantitative polymerase chain reaction (RT-qPCR).

**Discussion:**

Our study provides a comprehensive characterization of FA-MSCs, including for the first time MSC-GT and constitutes the largest series published to date. Interestingly, transcript profiling revealed dysregulation of metabolic pathways related to HSC self-maintenance. Taken together, our results or findings provide new insights into the pathophysiology of the disease, although whether these niche defects are involved in the hematopoietic defects seen of FA deserves further investigation.

## 1 Introduction

Fanconi anemia (FA) is a rare genetic disorder that results from DNA repair defects arising from pathogenic variants (PVs) in at least 22 genes (*FANCA*, *FANCB*, *FANCC*, *FANCD1/BRCA2*, *FANCD2*, *FANCE*, *FANCF*, *FANCG*, *FANCI*, *FANCJ/BRIP1*, *FANCL*, *FANCM*, *FANCN/PALB2*, *FANCO/RAD51C*, *FANCP/SLX4*, *FANCQ/ERCC4/XPF*, *FANCR/RAD51*, *FANCS/BRCA1*, FANCT/UBE2T, *FANCU/XRCC2*, *FANCV/REV7/MAD2L2*, and *FANCW/RFWD3*) discovered to play a role in the FA DNA repair pathway. All PVs in these genes are inherited in an autosomal recessive manner except those in *FANCB* and *FANCR/RAD51*, which are X-linked and autosomal dominant, respectively (Ceccaldi et al., 2016; Wegman-Ostrosky and Savage, 2017). Most patients with FA are characterized by bone marrow failure (BMF), somatic malformations, cancer predisposition and sensitivity to proinflammatory cytokines and alkylating agents (Svahn et al., 2016; Dufour and Pierri, 2022).

Mesenchymal stromal cells (MSCs) represent one of the key components of the bone marrow (BM) microenvironment, where they contribute to the creation of the hematopoietic stem cell (HSC) niche and play a crucial role in sustaining the development and differentiation of the hematopoietic system (Zhang et al., 2003). There is growing evidence suggesting that the microenvironment plays a role in several hematopoietic disorders, such as myeloproliferative and myelodysplastic neoplasms (Raaijmakers, 2012; Cogle et al., 2015; Li and Calvi, 2017; Curto-Garcia et al., 2020). However, the impact in the pathogenesis of BMF in FA remains unclear.

Few studies have described the role of the stroma in the hematological alterations of FA. It has been reported that the microenvironment could be involved in the pathogenesis of FA-related BMF in mice (Li et al., 2009). Also in mice, Zhou et al. have shown that MSCs have increased senescence, reduced proliferation and impaired differentiation, leading to skeletal alterations and a diminished ability to support hematopoiesis (Zhou et al., 2017). These data suggest that the pathogenesis of hematopoietic defects in FA is complex and likely depends, at least in part, on the interaction between abnormal hematopoietic cells and a dysfunctional niche. This hypothesis has only been analyzed in three studies with human samples. These studies described different alterations, some of them in a consistent manner, and others with controversial findings.

In this study we characterized BM-derived MSCs from pediatric patients affected by FA. We compared FA-MSCs with those expanded from healthy donors (HD-MSCs) to outline the differences that could elucidate the importance of the microenvironment on the exhaustion of HSC. Moreover, in patients that received a hematopoietic stem cell transplantation (HSCT) or gene therapy (GT), we studied MSCs before and after each treatment, in order to evaluate their potential impact on the BM niche.

## 2 Materials and methods

### 2.1 FA patients and healthy donors

Regarding inclusion criteria, pediatric patients with confirmed genetic diagnosis of FA assessed at the Hospital Infantil Universitario Niño Jesús between September 2018 and June 2021 could be included in the study. All patients with FA below 18 years that had undergone a bone marrow aspiration study to monitor their disease and had signed the informed consent were eligible for enrollment.

Some of the included patients did not receive treatment for bone marrow failure, while others were treated with HSCT or GT during their evolution. In those who received treatment, MSCs were isolated from BM aspirates obtained before and after treatment for the bone marrow failure.

As controls, we used MSCs isolated from HDs who underwent orthopedic surgery in which a bone marrow sample was obtained during the procedure.

This study was approved by the ethics committee of the Hospital Infantil Universitario Niño Jesus. Parents or legal guardians and HDs gave their written informed consent/assent.

### 2.2 Isolation and culture of BM-derived FA- and HD-MSCs

Mononuclear cells (MNCs) were isolated from BM aspirates (three to five mL) of FA patients and HDs by density gradient centrifugation and plated in non-coated 75–175 cm^2^ tissue culture flasks at a density of 500,000/cm^2^ in complete culture medium. MSCs were harvested, after reaching ≥80% confluence, using Trypsin, and were propagated at 5,000 cells/cm^2^.

### 2.3 Characterization of *ex-vivo* expanded FA- and HD- MSCs

#### 2.3.1 Immune-phenotype

Mesenchymal stromal cells were phenotypically characterized by flow-cytometry at P4 to evaluate the presence of the surface markers CD90, CD73 and CD29 and the absence of CD14, CD45 and CD19, using fluorescein isothiocyanate (FITC) or phycoerythrin (PE)-conjugated monoclonal antibodies (all from BioLegend and BD Biosciences). The sample was acquired on a FACSCanto II (BD) flow cytometer, and data were analyzed using the FACSDiva and Flowjo (BD) software.

#### 2.3.2 Proliferative capacity

Cell growth was analyzed by direct cell counts and population doublings (PDs) were determined at each passage. The number of PDs was calculated for each MSC sample by using the formula log_10_(N)/log_10_ (Wegman-Ostrosky and Savage, 2017) where N represents cells harvested/cells seeded; results were expressed as cumulative PD from passage (P) 1 to P5 (Zuk et al., 2001).

#### 2.3.3 Differentiation capacity

Adipogenic differentiation, osteogenic differentiation and the capacity to differentiate to cartilage tissue was evaluated as previously described (Mantelli et al., 2015). Differentiation was evaluated at P4–P6 by seeding MSCs at a density of 3.8 × 10^4^ in p12 plates for 6–7 days until 90% confluence. At that time, the medium was changed to the specific differentiation medium. After 15 days of culture differentiation was evaluated through the specific methodology in each case as reported in section 1.1 of the Supplementary Material.

#### 2.3.4 Fibroblast colony-forming unit (CFU-F) ability

CFU-F formation was assessed by examining the cultures at day +15; the clonogenic efficiency was calculated as the number of colonies per 6 × 10^3^MNCs initially seeded.

#### 2.3.5 Senescence assay

FA-MSCs and HD-MSCs were maintained in culture until reaching replicative senescence. MSCs were closely monitored during senescence for up to 20 passages before interrupting the cultures, in order to identify any change in morphology and/or proliferation rate. Senescence of MSCs was assessed by staining with β-galactosidase. Furthermore, senescence was also characterized among the samples included in the RNA-seq analysis by evaluating the transcriptomic data sets for genes and pathways associated with senescence.

### 2.4 MSC-mediated support of long-term hematopoiesis

The capacity of FA-MSC to support normal hematopoiesis *in vitro* was assessed as follows. Early passage (P4–P6) MSCs were irradiated (25 Gy by a Cesium irradiator) and plated into a 96-well plate at a concentration of 3 × 10^4^/well. One day later, CD34^+^ cells obtained by immunomagnetic selection of mobilized hematopoietic progenitors from HDs, were plated onto the MSC feeder at a concentration of 1 × 10^4^/well in the presence of Myelocult medium (StemCells Inc, Vancouver, BA, Canada) and 10^6^ mol/L hydrocortisone. The cultures were incubated for 5 weeks at 37°C, 5% CO_2_. Then, cultures were trypsinized and a classical methylcellulose assay was performed. The total number of colonies (colony-forming cells, CFCs) was scored after 14 days by an inverted microscope. Parallel experiments were performed using HD-MSCs as controls. Each experiment was performed in triplicates.

### 2.5 *In vitro* peripheral blood mononuclear cells (PBMNC) proliferation assay with phytohaemagglutinin

PBMNCs were obtained from peripheral blood samples from adult HDs. The proliferation of HD-PBMNCs in RPMI 1640 medium (Gibco, Life Technologies Ltd) supplemented with 10% FBS, in response to phytohaemagglutinin (PHA-P; Sigma- Aldrich), either in the presence or absence of MSCs, was performed in triplicate in flat-bottomed 96-well tissue culture plates (BD Falcon). Briefly, FA- and HD-MSCs were seeded at MSC:PBMNC ratios of 1:10 (10.000 MSC/100.000 PBMC) per well and allowed to adhere overnight before adding 1 × 10^5^ PBMNCs per well with or without PHA (4 lg/mL). After a 5-day incubation, the supernatant was collected for analysis by flow cytometry using CFSE (carboxyfluorescein succinimidyl ester) labeling. Lymphocyte proliferation (without MSC and stimulated by PHA) was considered as 100% proliferation and this percentage was used as a reference value to normalize or correlate lymphocyte proliferation in the presence of MSC.

### 2.6 Genetic characterization of FA-MSCs

#### 2.6.1 Mitomycin C (MMC) test

Due to the role of FA pathway proteins in DNA repair mechanisms, patient cells are extremely sensitive to DNA cross-linking agents such as MMC. MMC resistance testing was performed as previously described (González-Murillo et al., 2010; Diez et al., 2017). To assess this sensitivity, cells were exposed to increasing concentrations of MMC (0–333 nM; Sigma-Aldrich). The MSCs were seeded at a concentration of 5 × 10^3^ cells/cm^2^ in 24-well plates. 10–15 days afterwards cell viability was determined by flow cytometry with 7AAD.

#### 2.6.2 Chimerism studies

The chimerism study was carried out on MSC of patients with FA who were transplanted, to confirm whether the origin of these cells came from the donor or the recipient. This analysis was performed on DNA extracted from a MSC population at passage P4-P7 to avoid contamination with hematopoietic cells that could remain residual in the culture. The chimerism study was performed by microsatellite analysis, Short Tandem Repeats (STR), by PCR and fragment analysis. To quantify the levels of chimerism in the samples, the DNA profiles of the donor and recipient were previously characterized.

### 2.7 RNA-seq studies

#### 2.7.1 Ribonucleic acid preparation

For the study of the transcriptome, the cell fraction of the MSC cultures from P6-P8 was used. Total ribonucleic acid (RNA) from MSCs was extracted using Qiagen’s RNeasy Mini Kit, according to the manufacturer’s instructions. The total RNA that had a standard concentration of ≥200 ng/mL, mass ≥10 mg and RNA integrity number (RIN) ≥8.0 was subjected to RNA-Seq. Sequencing was performed at the Massive Sequencing Unit of the Madrid Science Park (NIMgenetics). The analysis was carried out by the Bioinformatics department of the Hospital Infantil Universitario Niño Jesús (section 1.2 of the Supplementary Material).

#### 2.7.2 Validation of gene expression by real time quantitative polymerase chain reaction (RT-qPCR)

To validate the results, the expression levels of seven selected transcripts were determined by RT-qPCR with the housekeeping gene GAPDH and RNA 18S as an endogenous reference. Relative quantification of the gene expression was determined normalizing the data of the gene to GAPDH and RNA 18S housekeeping gene and using the 2^−ΔΔCT^ method.

### 2.8 Statistical analysis

Quantitative variables were presented as the mean ± standard deviation (SD) or as the median ± range or interquartile range (IQR), as appropriate. All experiments were performed in triplicates.

The normal distribution was checked through the Saphiro-Wilk normality test. In cases where the samples did not demonstrate a normal distribution, comparisons were made using non-parametric tests. Thus, the Kruskal–Wallis’s test was used to compare means of >2 groups, while the Mann-Whitney test was used to compare means of two groups. When the results of normality test allowed it, parametric tests such as t-student or ANOVA were used to compare means. For comparisons of repeated measures, the Wilcoxon test was used in the case of two comparison groups. On the contrary, the Friedman test was used to compare repeated measures of >2 study groups. *p* values lower than 0.05 were considered to be statistically significant (*p < 0.05; **p < 0.01; ***p < 0.001). Statistical analysis was performed using SPSS Statistical Software version 22 and Graph Pad Prism 5.0 (Graph Pad Software, CA, EEUU).

## 3 Results

### 3.1 Patients and characteristics of the series

Twenty-eight FA pediatric patients assessed at the Hospital Infantil Universitario Niño Jesús between September 2018 and June 2021 were included in the study.

The median age at diagnosis was 4 years (range 1–12) and the median age at treatment was 6 years (range 2–14). 46.43% of the patients were male and 53.57% female. Diagnosis was confirmed by molecular studies in all cases. The vast majority of patients belonged to the complementation group A (*FANCA* 85.71%). Table 1 details patient characteristics.

**TABLE 1:** Patient characteristics. FA: Fanconi Anemia. HSCT: hematopoietic stem cell transplant. GT: Gene therapy. NA: not available.

	FA patients
N	28
Sex	
- Male, n (%)	13 (46.43)
- Female, n (%)	15 (53.57)
Age, years	
- At diagnosis, median (range)	4 (1–12)
- At treatment, median (range)	6 (2–14)
Diagnosis-treatment time	
- Years, median (range)	1 (0–6)
Complementation group	
- FANCA, n (%)	24 (85.71)
- FANCG, n (%)	3 (10.71)
- FANCD2, n (%)	1 (3.57)
Bone marrow failure severity	
- Severe, n (%)	3 (10.71)
- Moderate, n (%)	13 (46.42)
- Mild, n (%)	7 (25)
- NA, n (%)	5 (17.85)
Curative intent treatment for bone marrow failure	
- None, n (%)	7 (25)
- HSCT, n (%)	10 (35.71)
- GT, n (%)	11 (39.28)
Transfusion independent	
- Yes, n (%)	23 (82.14)
Patients alive	
- Yes, n (%)	27 (96.42)

We analyzed samples from twenty-one patients that received either HSCT or GT as curative intention treatment. HSCT was performed in 10 patients and 11 were treated within the GT trials (NCT03157804, NCT04248439). Eight patients underwent a non-related human leucocyte antigen (HLA)- matched donor transplant, whereas the remaining two children received an haploidentical T cell-depleted family donor HSCT and a matched related donor HSCT (embryo selection), respectively. In addition, we also analyzed samples from seven patients not receiving any treatment.

When feasible, MSCs were isolated from BM aspirates before and after treatment. The paired sequence of the same patient with a sample both prior and after treatment was achieved in 10 patients. Seven of them were treated with GT and three underwent HSCT. Within the follow-up of the patients in the gene therapy trial, marrow samples were obtained at the following time-points according to the trial protocol: at 6 months (n: 10), at 1 year (n:8) and beyond 1 year (n:8) from the infusion. However, no more than one replicate from the same patient was included in the same analysis.

As controls, we used MSCs isolated from 26 HDs with a median age of 17 years (range 13–21). Their peripheral blood cell counts were reviewed, confirming normality of the cell blood counts in all the cases.

Therefore, the total number of samples included in the study was 64, corresponding to 26 healthy donors, 28 FA patients and 10 paired samples of 10 of the FA patients.

### 3.2 Characterization of BM-derived FA-MSCs

MSCs were successfully expanded in all samples. Both FA-MSCs and HD-MSCs displayed the characteristic spindle-shaped morphology. All of them met the criteria established by the International Society for Cellular Therapy (ISCT) consortium, demonstrating >95% positivity for positive markers, and <2% for negative markers. HD-MSCs and FA-MSC prior and after treatment presented similar differentiation capacity.

**FIGURE 1:**
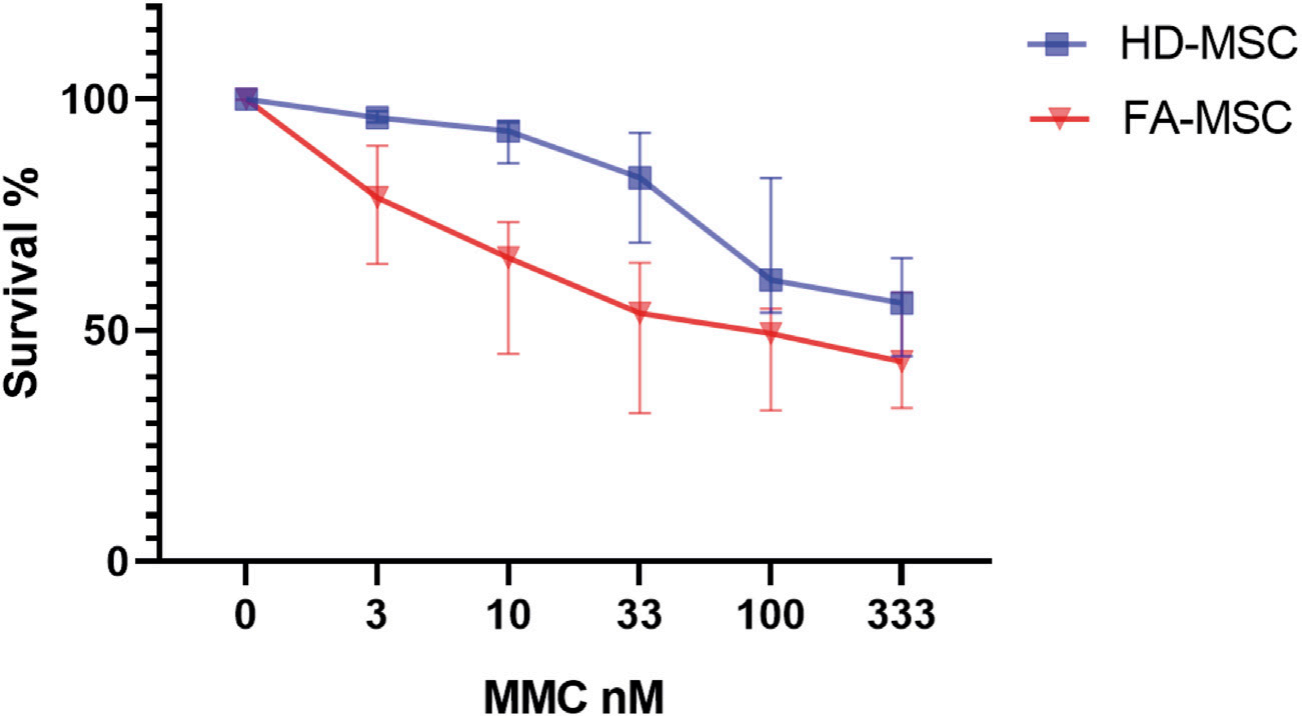
Mitomycin C (MMC) resistance testing. Cell viability and survival determined by flow cytometry with 7AAD at different MMC concentrations. Healthy donor’s mesenchymal stromal cells (HD-MSCs), Fanconi Anemia MSCs (FA-MSC).

MSC survival assays to DNA cross-linking agents confirmed that FA-MSCs cells were significantly more sensitive to MMC than HD-MSCs (*p* < 0.05) (Figure 1, and Supplementary Table S1). When performing the sub-analysis of the samples according to the study subgroups no differences were observed in survival (Supplementary Table S2). MSC chimerism was analyzed in nine of the patients who had received HSCT, demonstrating that the cellularity was 100% of the receptor, thus confirming the autologous origin of the stroma.

**FIGURE 2:**
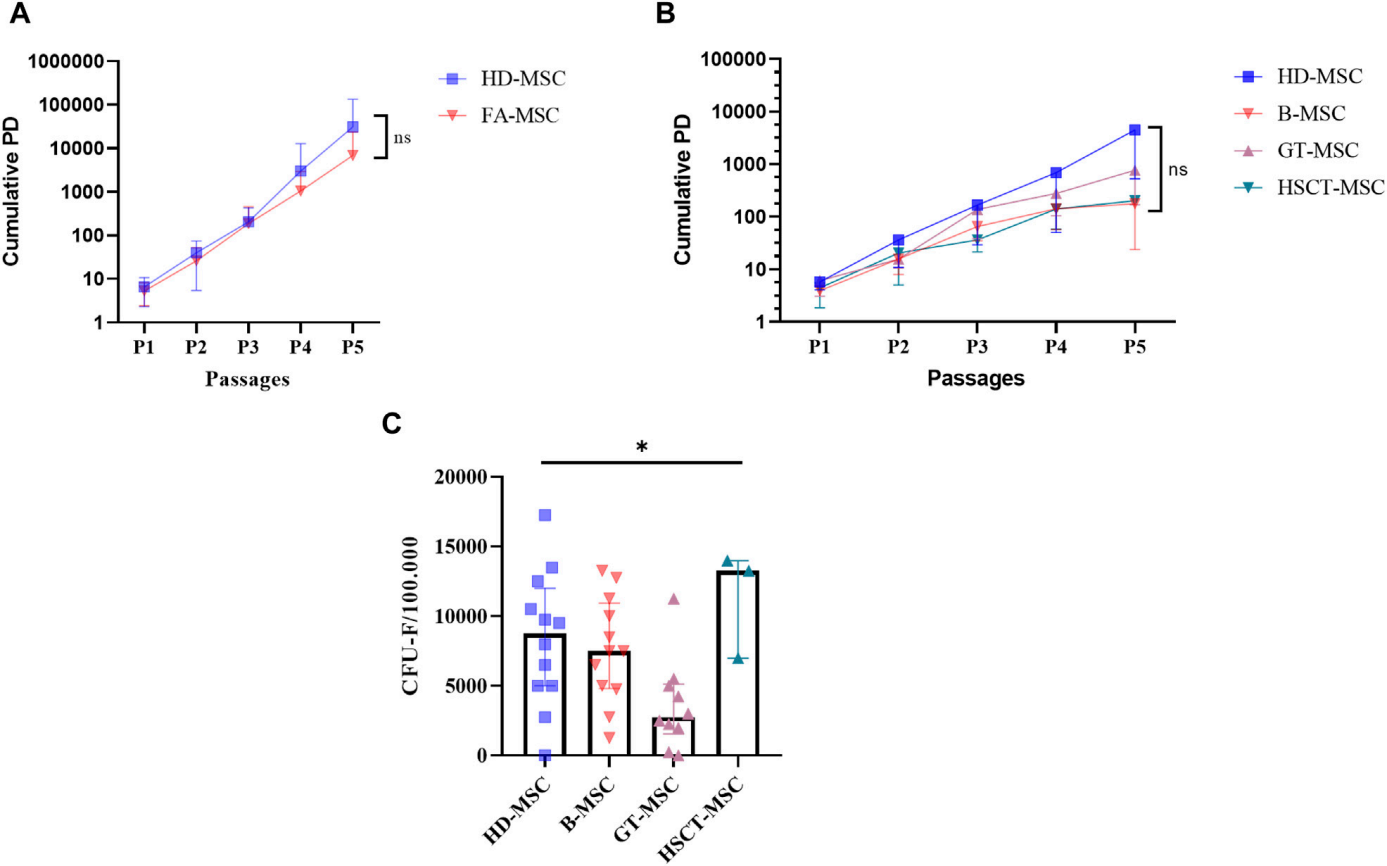
Proliferative capacity of mesenchymal stromal cells (MSCs). **(A)** Cumulative population doublings (PDs) from passage (P)1 to P5 of MSCs isolated from healthy donors (HDs) and from Fanconi Anemia (FA) patients. **(B)** Cumulative PDs from passage P1 to P5 of MSCs isolated from HDs and from FA patients divided into different subgroups: untreated or baseline-MSC (B-MSC), gene therapy-MSC (GT-MSC) and hematopoietic stem cell transplantation MSC (HSCT-MSC). **(C)** Fibroblast-colony forming unit (CFU-F) ability of FA-MSCs obtained before and after treatment (gene-therapy, GT-MSC or hematopoietic stem cell transplantation, HSCT-MSC) as compared with HD-MSCs.

In terms of proliferative capacity, there were no significant differences between FA-MSCs and HD-MSCs in early passages (Figure 2A, *p* = 0.38), nor among the different FA subgroups (Figure 2B, *p* = 0.38). Likewise, in seven paired samples of GT-MSC group no differences were observed before or after treatment (Supplementary Table S3). Interestingly, the same trend was confirmed in late cell culture passages where FA-MSCs presented a proliferative capacity similar to that of HD-MSCs (Supplementary Table S4). Thus, senescence was observed between P12-P19 for FA-MSC and between P11-P19 for HD-MSC (Supplementary Figure S1). These differences were not statistically significant (*p* = 0.85 in P19).

The clonogenic efficiency of FA-MSCs was comparable to that of HD-MSCs (6.45 vs. 8.35, *p* = 0.23). Of note, CFU-F ability of GT-MSCs was significantly lower than that of both HD- and FA-MSCs obtained at baseline or after HSCT (3.60 vs. 8.35 vs. 7.58 vs. 11.41, *p* = 0.015) (Figure 2C). Six paired samples from three patients undergoing gene therapy were then analyzed. The three post-treatment samples demonstrated a trend towards a reduced capacity to generate CFU-F compared to the pre-treatment samples (10.00 vs. 2.50) (Supplementary Table S5).

### 3.3 Ability of FA-MSCs to support long-term hematopoiesis

**FIGURE 3:**
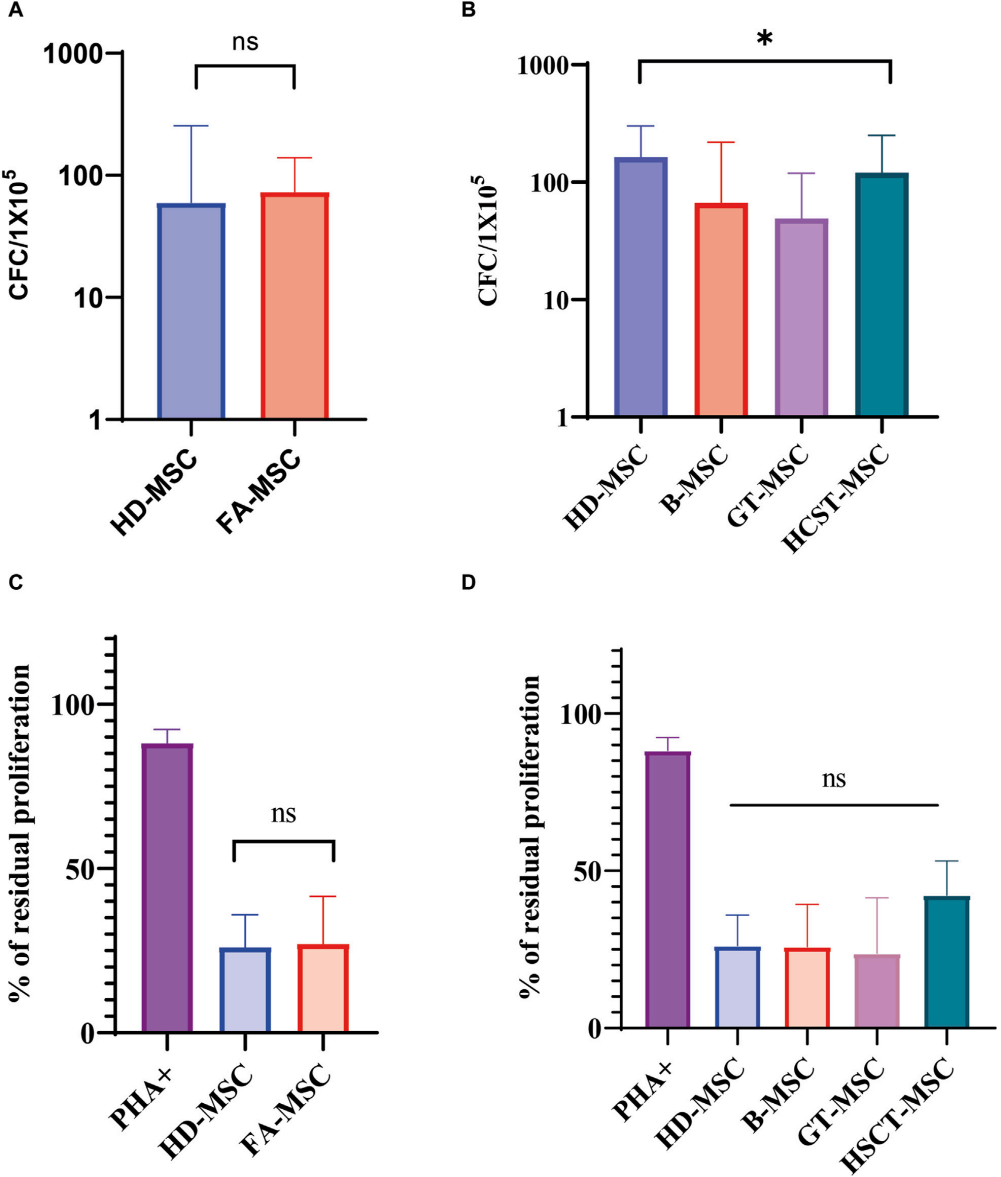
Functional characterization of mesenchymal stromal cells (MSCs). **(A)** Ability of Fanconi Anemia (FA) MSCs (FA-MSCs) and healthy donors MSC (HD-MSC) to support long-term hematopoiesis. Results are expressed as the median number of colony-forming-cells (CFCs) and represent the median of triplicate experiments. Ns: non-significant. **(B)** Ability to support long-term hematopoiesis of the different study subgroups: HD-MSCs, baseline or untreated FA patients (B-MSCs), and FA patients that received gene therapy (GT-MSCs) or hematopoietic stem cell transplant (HSCT-MSCs). Results are expressed as the median number of CFCs and represent the median of triplicate experiments. **(C)** *In vitro* immunomodulatory effect of HD-MSCs and FA-MSCs on peripheral blood mononuclear cells (PBMNCs) in an allogeneic setting. The graph shows the percentage of residual proliferation of PBMNCs stimulated with phytohaemagglutinin (PHA) in the presence of HD-MSCs or FA-MSCs. Each bar represents the percentage of residual proliferation of 10^5^ PBMNCs, in the presence of MSC:PBMNC at a ratio of 1:10. **(D)** *In vitro* immunomodulatory effect of the different study subgroups on PBMCs in an allogeneic setting. The graph shows the percentage of residual proliferation of PBMCs stimulated with phytohaemagglutinin (PHA) in the presence of HD-MSCs, B-MSC, GT-MSC or HSCT-MSC. Each bar represents the percentage of residual proliferation of 10^5^ PBMCs, in the presence of MSC:PBMC at a ratio of 1:10.

The capacity to support hematopoiesis was evaluated in 14 FA patients and 3 HDs at early passages. Our results showed that the CFC output of long-term culture-initiating cell (LTC-IC) assays did not significantly differ between MSCs derived from HDs and those from FA patients (Figure 3A).

Nonetheless, the ability to maintain hematopoiesis was not equal when comparing the four study subgroups (HD-MSC, B-MSC, GT-MSC, and HSCT-MSC, *p* = 0.016) suggesting that the generation of CFC was lower in the GT-MSC group (Figure 3B and Supplementary Table S6). When analyzing four of the patients belonging to the GT group in a paired manner, the ability to support hematopoiesis did not seem to be determined by treatment, since CFC generation was similar before and after GT (6.35 vs. 6.88, respectively) (Supplementary Table S7).

### 3.4 Effect of FA-MSCs on PHA-induced PBMNC proliferation

We measured PBMNC proliferation induced by PHA either in the presence or in the absence of MSCs. 37 samples were analyzed: five of them corresponding to HD-MSC, 15 MSC from patients without treatment (B-MSC), and 17 MSC samples from patients treated for their BMF (11 GT-MSC and 6 HSCT-MSC).

FA-MSC exerted an inhibitory effect on PHA-induced PBMNC proliferation similar to HD-MSC in the 1:10 ratio, showing a median proliferation percentage of 27.65% (IQR 17.23–42.32) *versus* 26.19% (IQR 15.14–35.94), respectively (*p* = 0.27) (Figure 3C). Of note, HSCT-MSC showed a trend towards a greater residual proliferation in comparison to the other study groups, but this difference was not statistically significant (B-MSC 25.84% [IQR 16.82–39.30], GT-MSC 25.84% [IQR 16.82–39.30], and HSCT-MSC 42.23 [20.54–53.13], *p* = 0.13) (Figure 3D). Overall, these results indicate that MSCs isolated from the BM of FA patients have a similar immunomodulatory effect as MSCs from HDs, and that it is not significantly affected by treatment.

### 3.5 Transcriptomic profiling of FA patients (FANCA −/−) *versus* healthy donors MSCs

We compared the transcriptomic profiling of seven untreated FA-MSC samples and 3 HD-MSCs. All seven patients had a pathogenic variant in the *FANCA* gene.

**FIGURE 4:**
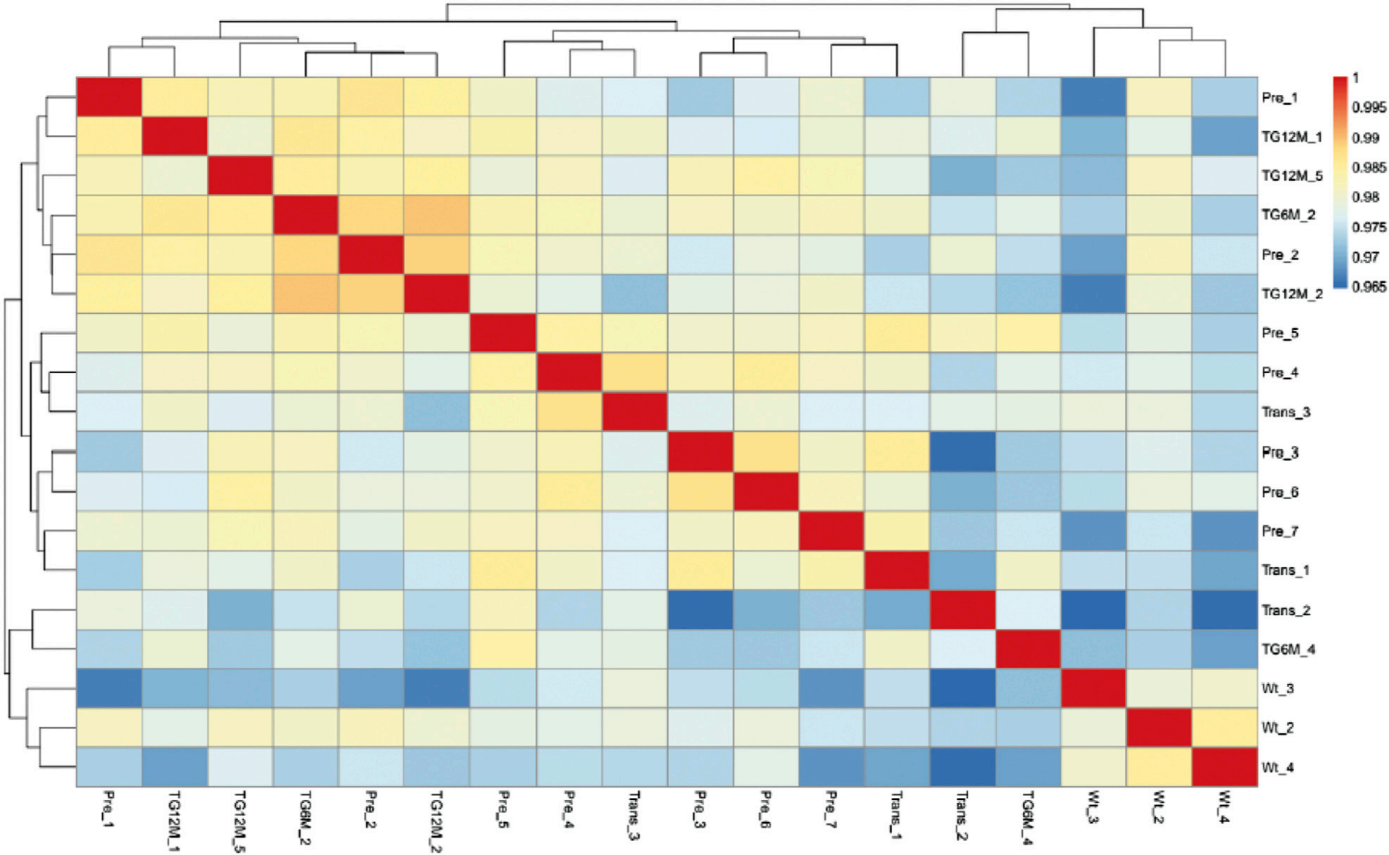
Transcript profiling of Fanconi Anemia (FA) patients *versus* healthy donors (HD) mesenchymal stromal cells (MSCs). Heat-map illustration of gene expression FA-MSCs from FA patients (n = 7) and HD-MSCs (n = 3).

RNA-seq expression profiling of FA-MSC *versus* HD-MSC segregated populations based on hierarchical clustering (Figure 4). Principal-component analysis revealed two differentiated groups, according to the FA pathway, three samples of healthy donors on the one hand, and the group of patients on the other.

**FIGURE 5:**
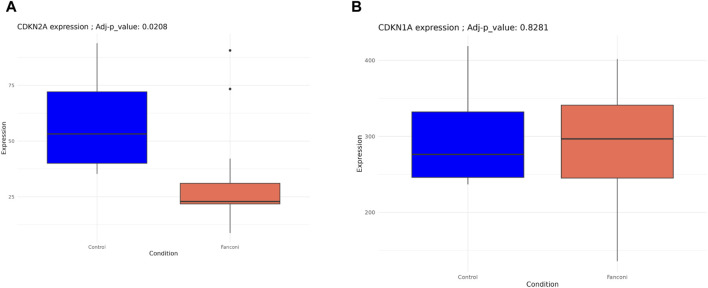
Transcript profiling of senescence-associated pathways from FA-MSCs (n = 7) and HD-MSCs (n = 3). **(A)** CDKN2A/p16 gene expression FA-MSCs from FA patients (n = 7) and HD- MSC (n = 3). **(B)** CDKN1A/p21 gene expression FA-MSCs from FA patients (n = 7) and HD- MSC (n = 3).

To evaluate whether the exhaustion of HSCs is related to the chronic activation of stress signaling pathways and to conclude about its effect in cell-aging or senescence process, we conducted a targeted analysis to evaluate some of the master regulators of senescence, as well as senescence-associated secretory phenotype (SASP). Among these senescence hallmarks, only CDKN2A/p16 differed significantly between FA-MSC and HD-MSC, so that HD-MSCs showed an overexpression CDKN2A/p16 (p 0.02, Figure 5A). However, FA-MSCs showed a trend to a higher repression in pathways such as CDKN1A/p21 (Figure 5B) or SASP complex (IL6, IL1-α, IGF-BP3), without reaching statistical significance in any of the cases.

Besides, as it is known that MSC-derived secretomes contribute to activating an inflammatory transcriptome, we also evaluated other pathways related to inflammation. Indeed, no significant dysregulations were observed between groups regarding other inflammation-related pathways such as NF-KB, SMAD2/3, TGF-β or TNF-α.

**FIGURE 6:**
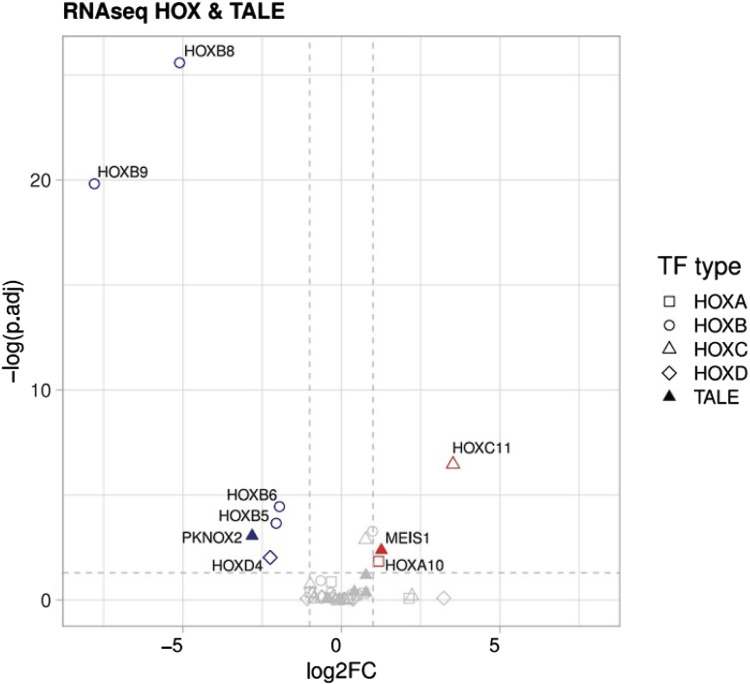
Transcriptomic studies. Differences between downregulated vs. upregulated pathways in HOX and TALE members.

Finally, we performed a targeted analysis to investigate the expression of HOX and TALE transcription factors as noted by a previous group in the FA-MSC setting. (Cagnan et al., 2019). It is worth mentioning that our analysis identified repression of *HOXB 5-6-eight to nine* and *HOXD4*, with overexpression of *HOXA10* and *HOXC11* (Figure 6). Moreover, due to the fact that the HOX pathway is one of the driving mechanisms of cancer development such as leukemia we evaluated the genes related to its evolution. However, no alterations were identified in FA-MSC in the genes related to myelodysplasia/acute leukemia.

**Validation of the sequencing results by RT-qPCR.** To confirm the reliability of the expression profiles generated using the RNA-Seq and DEGs analysis, RT-qPCR was applied in seven up/downregulated candidate genes (*HOXC 11, HOXA 10, HOXD 4, HOXB 5, HOXB 6, HOXB 8*, and *HOXB 9*). As expected, the RT-qPCR results matched the RNA-seq results in the majority, except in *HOXA 10* gene, in which its overexpression was not confirmed in more than half of the samples.

## 4 Discussion

Despite the genetic heterogeneity of patients with FA, many of them share a common phenotype characterized by the development of BMF. It is estimated that the risk of BMF is 50% at the age of 40, and in 75% of the cases it occurs during the first decade of life (Alter, 2014; Alter et al., 2022). Thus, most of the therapies for these patients are focused on treating BMF.

Nonetheless, the pathophysiology of the exhaustion of HSCs remains unclear. There is increasing evidence suggesting a potential role of the microenvironment as a contributing component in other hematopoietic disorders (Cogle et al., 2015; Curto-Garcia et al., 2020). In the present study, we expanded *ex vivo* MSCs derived from BM of children with FA, both before and after receiving treatment and compared with those obtained from HDs, in order to address whether the niche plays a role in the BMF of these patients.

Prior to our study, the first group that explored the involvement of the niche in the BMF of human FA patients included only samples from untreated subjects. They found functional deficits in MSC after long-term cultures, concluding that FA-MSC could be involved in the development of BMF (Lecourt et al., 2010). Later, Mantelli et al. expanded this information with the characterization of FA-MSC of patients treated with HSCT (Mantelli et al., 2015). They pointed out that FA-MSC were defective in their ability to proliferate, but they maintained their functionality as HD-MSC. Hence, they could not conclude that these phenotypic defects impacted on the pathophysiology of BMF. To the best of our knowledge, the present work is the largest series published to date with MSCs from FA patients. Furthermore, we characterized the stroma of patients treated with GT for the first time, not only in FA but also in other monogenic diseases where gene therapy is being used.

Our data showed that FA-MSCs exhibit similar morphology, immunophenotype and differentiation potential to HD-MSCs as in the previous studies (Lecourt et al., 2010; Mantelli et al., 2015).

MMC survival assays demonstrated that FA-MSCs cells were significantly more sensitive to DNA cross-linking agents in comparison to HD-MSCs confirming that the FA pathway is not functional in stromal cells either. Moreover, according to other groups, when comparing MSCs after transplant we confirmed the autologous origin of the stroma. In HSCT recipients, MSCs remain of recipient origin, indicating that these cells are not fully eradicated by a non-myeloablative conditioning.

In accordance with Mantelli et al, the proliferative capacity of FA-MSCs obtained before treatment was comparable to that of HD-MSCs. However, in contrast to what was observed by Mantelli`s group, in our study FA-MSC obtained after HSCT did not display a significantly lower proliferative capacity compared to HD-MSCs. As we will discuss later on, the time of collection of the samples after transplant could influence these results.

When evaluating the proliferative capacity at high passages of cell cultures (P5-P19) and the *in vitro* life-span of FA-MSCs, we found that these cells obtained before and after HSCT or GT did not develop signs of senescence earlier than HD-MSCs, as opposed to the previously reported data (Lecourt et al., 2010; Mantelli et al., 2015). This difference may be due to the duration of the cultures, since Lecourt et al. reported a median culture time of 11–12 weeks, Mantelli et al. pointed out a duration between 8 and 12 weeks, and in our series the majority were between 7 and 8 weeks.

Regarding clonogenic efficiency, in line with previously reported data, we did not find any major differences in fibroblast colony-forming unit ability between B-MSC and HD-MSC. However, in contrast to what was observed by Mantelli et al(Mantelli et al., 2015), in our series FA-MSC obtained after HSCT did not display a significantly lower clonogenic efficiency compared to HD-MSCs. They suggested that the differences they observed might indicate either an intrinsic defect of FA-MSCs or a toxic effect of the conditioning regimens (Mantelli et al., 2015). In our opinion, the disparity of our results in this respect, and those related to the early proliferative capacity, compared to those of Mantelli´s can be explained by the time elapsed from the treatment to the obtaining of the sample for the clonogenic efficiency analysis. Mantelli’s group evaluates the cohort of post-HSCT samples 100 days after treatment, whereas in our series the median time was greater than 12 months. It has been previously described that these differences in the time of collection of the samples could be critical in order to interpret the results. Ding et al, showed that the amount of MSC was drastically reduced in the early phase after HSCT and returned to a normal level 9 months after transplantation. Thus, the number of CFU-F increases in a time-dependent manner from the first month and reaches approximately 90% in the ninth month after HSCT (Ding et al., 2014). Also of note was our observation that the MSCs obtained after GT showed a trend to a lower clonogenic capacity. Nevertheless, the limited number of samples prevents us from clearly concluding on its functional inferiority.

It is worth to note that, the subgroup of MSC-GT showed a tendency to generate less CFU-F than the other subgroups. However, these data should be evaluated with caution due to the scarce number of samples. Moreover, the abscense of this type of studies in the GT setting does not allow us to support our results with the experience of other groups.

On the question of whether there were differences in the functionality of the MSCs, in line with previously reported data, we did not find any major differences in the ability to support long-term hematopoiesis between FA-MSCs and HD-MSCs, suggesting that FA- MSCs do not display, *in vitro* and at early passages, an impaired capacity to sustain the proliferation and expansion of HD-HSCs (Mantelli et al., 2015). We also investigated the ability of FA-MSC to inhibit *in vitro* mitogen-induced PBMNC proliferation, both before and after treatment, not observing statistically significant differences between groups. In summary, in accordance with Mantelli et al’s work, these data indicate that the main functions of the MSCs are globally preserved in FA patients (Mantelli et al., 2015).

In addition, we performed a thorough analysis of molecular pathways related to different processes in the niche. Bone marrow failure in FA is multifactorial and largely results from the death of HSCs due to genomic instability. Following DNA damage, proteins of the FA pathway act in a complex cascade to repair interstrand crosslinks, which are caused by reactive oxygen species or exposure to reactive aldehydes. Moreover, the elevation of inflammatory markers and hypersusceptibility to proinflammatory cytokines that induce cell death is a phenotype associated with FA gene mutations. In this respect, Oppezzo et al demonstrated the association between bone marrow failure and the constitutive expression of Microphthalmia Transcription Factor (MiTF) in mouse FA HSCs, through the cooperative, unscheduled activation of several stress-signaling pathways, including the SMAD2/3, p38 MAPK, NF-κB, and AKT cascades (Oppezzo et al., 2020). A growing amount of evidence has demonstrated that the activation of these pathways leads to a state of quiescence in HSCs, and therefore to the accumulation of DNA damage and a reduction in their repopulation capacity (De Haan and Lazare, 2018; Di Micco et al., 2021). This supports the hypothesis that the exhaustion of HSCs in FA is the consequence of defects in the response to DNA damage combined with the chronic activation of stress signaling pathways that in a normal situation should be activated only transiently.

However, as the mentioned studies reflect, this inflammatory background in FA has been previously studied in HSCs, hence the impact of the niche is still not clear. We studied pathways related to inflammation in our MSC samples, such as NF-κB, SMAD 2/3, IL-6, TGF-β or TNF-α, and have not found dysregulation in any of them. Therefore, this suggests that the constitutive expression is intrinsic to the HSCs but not to the stroma cells.

As demonstrated by others, inflammation is tightly related to cellular aging or senescence. Even though the processes behind MSC senescence remain unclear, several studies have made progress in elucidating the aspects of the age-related changes (Weng et al., 2022). Stenderup et al found out that MSCs from older donors exhibited accelerated senescense comparing with MSCs from younger donors (aged 18–29 years *versus* aged 68–81 years) (Stenderup et al., 2003). Unfortunately, no similar studies have been published with pediatric cohorts to our knowledge.

When we expanded the characterization of MSC senescence in our samples by reviewing several hallmarks or master regulators of a senescent phenotype, CDKN2A/p16 was the only one that was differentially expressed among the two study groups, showing an overexpression in HD-MSCs. This finding suggests that MSCs from our FA patients are less senescent than the HD-MSCs included in the study. Nevertheless, we did not observe differences in other genes or pathways such as CDKN1A/p21 or senescense-associated secretory phenotype (SASP) complex, nor in long-term cultures or in β-galactosidase staining assay either, suggesting that there are no major differences in the cellular ageing between both groups.

Finally, we performed a targeted analysis to investigate the expression of HOX and TALE transcription factors in FA-MSC. It is thought that these are important regulators of development and homeostasis, determining cellular identity and predisposing to cancer progression if dysregulated (Cagnan et al., 2019). Cagnan et al, compared the expression levels of those genes in bone marrow MSCs obtained from FA patients and HDs. In general, they observed highly conserved expression levels between patient and donor cells, except in PKNOX2 which was downregulated. In contrast, our transcriptomic studies did not find differences in PKNOX2 expression between FA-MSC patients and HDs. Conversely, we found differences among several HOX members, which were not observed by Cagnan et al. (Cagnan et al., 2019) Nevertheless, methodological differences should be kept in mind. It would be intringuing to compare expression changes of these genes upon cell passing, since Cagnan et al used MSCs at the third culture passage to analyze the differential expression whereas we used MSCs at passage six.

It is worth mentioning that our study has several limitations. First, age was not homogeneous between the two main study groups (FA-MSC and HD-MSC). The median age in HD was significantly higher since usually the patients that undergo orthopedic surgery are teenagers, whereas the diagnose of FA is usually made before age 10 years. Despite this, we decided to use this population as a control group, given the difficulty of accessing BM samples from younger HD. In our opinion, the age difference between both groups should not affect the phenotype and functionality of the MSCs in a pediatric cohort. For instance, in the senescence studies, we found no major differences between both groups, although differences had been previously reported related with age (Stenderup et al., 2003). However, the age range between both cohorts of that study (18–21 *versus* 68–81) is much wider than the difference between the patients and HDs included in ours, which together with the fact that no other data were found analyzing samples from pediatric patients in this or other diseases, does not allow us to establish an age threshold related to the MSC features.

Second, the absence of similar studies in GT settings makes the interpretation of some of the particularities of that MSC subgroup more challenging. Third, transcriptomic studies have been carried out on a small number of samples. In fact, samples from treated patients were not included. However, these results must be evaluated considering the scenario of a pediatric rare disease in which obtaining a sufficient sample for several studies is not always feasible.

In conclusion, our study provides a comprehensive characterization of BM-derived MSCs obtained from FA patients, including for the first time MSCs from patients treated with gene therapy. Our findings suggest that FA-MSCs maintain their main functional properties, making their direct implication in BMF development less likely. Interestingly, a suppressed metabolic pathway has been identified in FA-MSC (HOX). In this sense, more studies will be needed to further explore the possible involvement of the niche in the evolution of bone marrow failure of FA patients.

## Data Availability

The data presented in the study are deposited in a public repository: https://www.ncbi.nlm.nih.gov/bioproject/1114677. Submission ID: SUB14594881, BioProject ID: PRJNA1114677.

## References

[B1] Alter B. P. (2014). Fanconi anemia and the development of leukemia. Best. Pract. Res. Clin. Haematol. 27 (3–4), 214–221. 10.1016/j.beha.2014.10.002 25455269 PMC4254647

[B2] Alter B. P. Giri N. McReynolds L. J. Altintas B. (2022). Fanconi anaemia: a syndrome with distinct subgroups. Br. J. Haematol. 197 (4), 467–474. 10.1111/bjh.18091 35191533 PMC11844804

[B3] Cagnan I. Cosgun E. Konu O. Uckan D. Gunel-Ozcan A. (2019). PKNOX2 expression and regulation in the bone marrow mesenchymal stem cells of Fanconi anemia patients and healthy donors. Mol. Biol. Rep. 46 (1), 669–678. 10.1007/s11033-018-4522-z 30515693

[B4] Ceccaldi R. Sarangi P. D’Andrea A. D. (2016). The Fanconi anaemia pathway: new players and new functions. Nat. Rev. Mol. Cell. Biol. 17 (6), 337–349. 10.1038/nrm.2016.48 27145721

[B5] Cogle C. R. Saki N. Khodadi E. Li J. Shahjahani M. Azizidoost S. (2015). Bone marrow niche in the myelodysplastic syndromes. Leuk. Res. 39 (10), 1020–1027. 10.1016/j.leukres.2015.06.017 26276090

[B6] Curto-Garcia N. Harrison C. McLornan D. P. (2020). Bone marrow niche dysregulation in myeloproliferative neoplasms. Haematologica 105 (5), 1189–1200. 10.3324/haematol.2019.243121 32241851 PMC7193484

[B7] De Haan G. Lazare S. S. (2018). Aging of hematopoietic stem cells. Blood 131 (5), 479–487. 10.1182/blood-2017-06-746412 29141947

[B8] Diez B. Genovese P. Roman-Rodriguez F. J. Alvarez L. Schiroli G. Ugalde L. (2017). Therapeutic gene editing in CD34(+) hematopoietic progenitors from Fanconi anemia patients. EMBO Mol. Med. 9 (11), 1574–1588. 10.15252/emmm.201707540 28899930 PMC5666315

[B9] Di Micco R. Krizhanovsky V. Baker D. d’Adda Di Fagagna F. (2021). Cellular senescence in ageing: from mechanisms to therapeutic opportunities. Nat. Rev. Mol. Cell. Biol. 22 (2), 75–95. 10.1038/s41580-020-00314-w 33328614 PMC8344376

[B10] Ding L. Zhu H. Yang Y. Wang Z. D. Zheng X. L. Yan H. M. (2014). Functional mesenchymal stem cells remain present in bone marrow microenvironment of patients with leukemia post-allogeneic hematopoietic stem cell transplant. Leuk. Lymphoma 55 (7), 1635–1644. 10.3109/10428194.2013.858815 24180332

[B11] Dufour C. Pierri F. (2022). Modern management of Fanconi anemia. Hematol. Am. Soc. Hematol. Educ. Program 2022 (1), 649–657. 10.1182/hematology.2022000393 PMC9821189 36485157

[B12] González-Murillo Á. Lozano M. L. Álvarez L. Jacome A. Almarza E. Navarro S. (2010). Development of lentiviral vectors with optimized transcriptional activity for the gene therapy of patients with Fanconi anemia. Hum. Gene Ther. 21 (5), 623–630. 10.1089/hum.2009.141 20001454

[B13] Lecourt S. Vanneaux V. Leblanc T. Leroux G. Ternaux B. Benbunan M. (2010). Bone marrow microenvironment in Fanconi anemia: a prospective functional study in a cohort of Fanconi anemia patients. Stem Cells Dev. 19 (2), 203–208. 10.1089/scd.2009.0062 19572808

[B14] Li A. J. Calvi L. M. (2017). The microenvironment in myelodysplastic syndromes: niche-mediated disease initiation and progression. Exp. Hematol. 55, 3–18. 10.1016/j.exphem.2017.08.003 28826860 PMC5737956

[B15] Li Y. Chen S. Yuan J. Yang Y. Li J. Ma J. (2009). Mesenchymal stem/progenitor cells promote the reconstitution of exogenous hematopoietic stem cells in Fancg−/− mice *in vivo* . Blood 113 (10), 2342–2351. 10.1182/blood-2008-07-168138 19129541 PMC2742339

[B16] Mantelli M. Avanzini M. A. Rosti V. Ingo D. M. Conforti A. Novara F. (2015). Comprehensive characterization of mesenchymal stromal cells from patients with Fanconi anaemia. Br. J. Haematol. 170 (6), 826–836. 10.1111/bjh.13504 26010568

[B17] Oppezzo A. Bourseguin J. Renaud E. Pawlikowska P. Rosselli F. (2020). Microphthalmia transcription factor expression contributes to bone marrow failure in Fanconi anemia. J. Clin. Investig. 130 (3), 1377–1391. 10.1172/JCI131540 31877112 PMC7269568

[B18] Raaijmakers MHGP (2012). Myelodysplastic syndromes: revisiting the role of the bone marrow microenvironment in disease pathogenesis. Int. J. Hematol. 95 (1), 17–25. 10.1007/s12185-011-1001-x 22218882

[B19] Stenderup K. Justesen J. Clausen C. Kassem M. (2003). Aging is associated with decreased maximal life span and accelerated senescence of bone marrow stromal cells. Bone 33 (6), 919–926. 10.1016/j.bone.2003.07.005 14678851

[B20] Svahn J. Bagnasco F. Cappelli E. Onofrillo D. Caruso S. Corsolini F. (2016). Somatic, hematologic phenotype, long-term outcome, and effect of hematopoietic stem cell transplantation. An analysis of 97 Fanconi anemia patients from the Italian national database on behalf of the Marrow Failure Study Group of the AIEOP (Italian Association of Pediatric Hematology-Oncology). Am. J. Hematol. 91 (7), 666–671. 10.1002/ajh.24373 27013026

[B21] Wegman-Ostrosky T. Savage S. A. (2017). The genomics of inherited bone marrow failure: from mechanism to the clinic. Br. J. Haematol. 177 (4), 526–542. 10.1111/bjh.14535 28211564

[B22] Weng Z. Wang Y. Ouchi T. Liu H. Qiao X. Wu C. (2022). Mesenchymal stem/stromal cell senescence: hallmarks, mechanisms, and combating strategies. Stem Cells Transl. Med. 11 (4), 356–371. 10.1093/stcltm/szac004 35485439 PMC9052415

[B23] Zhang J. Niu C. Ye L. Huang H. He X. Tong W. G. (2003). Identification of the haematopoietic stem cell niche and control of the niche size. Nature 425 (6960), 836–841. 10.1038/nature02041 14574412

[B24] Zhou Y. He Y. Xing W. Zhang P. Shi H. Chen S. (2017). An abnormal bone marrow microenvironment contributes to hematopoietic dysfunction in Fanconi anemia. Haematologica 102 (6), 1017–1027. 10.3324/haematol.2016.158717 28341737 PMC5451333

[B25] Zuk P. A. Zhu M. Mizuno H. Huang J. Futrell J. W. Katz A. J. (2001). Multilineage cells from human adipose tissue: implications for cell-based therapies. Tissue Eng. 7 (2), 211–228. 10.1089/107632701300062859 11304456

