# Supplementary material for: Role of the mesenchymal stromal cells in bone marrow failure of Fanconi Anemia patients
Source: Front Cell Dev Biol. 2024 Jul 25;12:1286815. doi: 10.3389/fcell.2024.1286815 (PMC11307092; doi:10.3389/fcell.2024.1286815)
Supplement: Supplementary file 1 [file DataSheet1.docx]

**Supplementary Material**

**1. Material and Methods**

**1.1 Characterization of ex-vivo expanded FA- and HD- MSCs**

**Differentiation capacity.** Adipogenic differentiation, osteogenic differentiation and the capacity to differentiate to cartilage tissue was evaluated as previously described^19^. Differentiation was evaluated at P4–P6 by seeding MSCs at a density of 3.8x10^4^ in p12 plates (BD, Falcon) in DMEM (DMEM 1g/L glucose, Gibco, Carlsbad, CA), supplemented with 10% fetal bovine serum (FBS, HyClone, Logan, UT) for 6 to 7 days until 90% confluence. At that time, the medium was changed to the specific differentiation medium. Adipogenic differentiation was evaluated at P4–P6 by seeding MSCs at a density of 3.8x10^4^ in p12 plates (BD, Falcon) in DMEM (DMEM 1g/L glucose, Gibco, Carlsbad, CA), supplemented with 10% fetal bovine serum (FBS, HyClone, Logan, UT) for 6 to 7 days until 90% confluence. At that time, the medium was changed to the specific differentiation medium (StemPro Adipogenesis Differentiation Kit, GIBCO) composed of α-MEM supplemented with 4.5g/L D-glucose, 110mg/L sodium pyruvate, Glutamax, and 1ml Penicillin P/S. The differentiation medium was renewed every 3 days and, after 15 days of culture adipogenic differentiation was evaluated through the morphological appearance of fat droplets stained with Oil Red O (Sigma-Aldrich). The osteogenic differentiation capacity of FA-MSCs was assessed at P4–P6 by incubating cells with a minimal essential medium (StemPro Osteogenesis Differentiation Kit, GIBCO) composed of 1g/L D-Glucose 110mg/L, sodium phosphate, Glutamax, 1ml Penicillin P/S starting from day +7 of the culture. The medium was renewed every 3 days and on day 15 of the culture, calcium deposition was evaluated with Alizarin Red (Sigma-Aldrich). Both osteogenic and adipogenic cultures were incubated for at least 2 weeks before evaluating differentiation. To determine the differentiation capacity of MSCs to cartilage tissue, MSCs from P4-P6 were plated with the specific differentiation medium (StemPro chondrogenesis differentiation kit, GIBCO) composed of 1g/L D Glucose, 110mg/L sodium pyruvate, Glutamax, 1ml Penicillin P/S. The medium was changed every 3 days and staining with Alcian Blue was carried out on day 15-20 of the culture to evaluate the differentiation. This technique is used to determine the presence of acid mucopolysaccharides in cartilage sections and cell cultures. It is based on staining the sulfoxide groups of glycans blue by staining with Alcian Blue (Alcian Blue, Sigma-Aldrich).

**1.2 RNA-seq studies**

**Ribonucleic acid preparation.** For the study of the transcriptome, the cell fraction of the MSC cultures from P6-P8 was used. Total ribonucleic acid (RNA) from MSCs was extracted using Qiagen's RNeasy Mini Kit, according to the manufacturer’s instructions. RNA samples were then digested with RNase free DNase I (Invitrogen, Carlsbad, CA, USA) to eliminate residual genomic deoxyribonucleic acid (DNA), and the digestion products were purified using magnetic beads (Axygen, Union City, CA, USA). Lastly, the concentration, quality and integrity of the total RNA were determined using a Nano Drop 2000 spectrophotometer (Thermo Scientific, Wilmington, DE, USA) and an Agilent 2100 Bioanalyser (Agilent Technologies, Santa Clara, CA, USA). The total RNA that had a standard of concentration ≥200 ng/mL, mass ≥10 mg and RNA integrity number (RIN) ≥8.0 was subjected to RNA-Seq. Each sample was sent to the Massive Sequencing Unit of the Madrid Science Park (NIMgenetics, New Integrated Medical Genetics) where sequencing was performed. The generation of libraries, as well as the steps prior to the analysis of differential expression, were carried out by a subcontracted outsourced service (NIMgenetics). The analysis of the differential expression was carried out by the Bioinformatics service of the Hospital Infantil Universitario Niño Jesús.

**Complementary deoxyribonucleic acid library construction.** Messenger (m)RNA for each sample was enriched using oligo (dT) magnetic beads and then fragmented into short pieces (approximately 200 bp) in fragmentation buffer. The first strand cDNA was transcribed from the cleaved RNA fragments using random hexamer primer, and second strand complementary (c)DNA was synthesised in a reaction containing buffer, dNTPs (deoxyribonucleotide triphosphate), RNase H and DNA polymerase I. Double stranded cDNA was purified with magnetic beads and subjected to end reparation and 3^´^ single adenylation. Integrity and size were checked on an Agilent 2100 Bioanalyser (Agilent technologies, Santa Clara, CA, USA). Lastly, the cDNA library was sequenced on a paired end flow cell using an Illumina Hiseq^TM^ 4000 platform (Illumina, San Diego, CA, USA).

**Ribonucleic acid sequencing data processing and identification of differentially expressed genes.** cDNA libraries from MSCs were sequenced according to the protocols for RNA-Seq. Raw reads were pre-processed using FastQC software. PCR duplicates, reads that only contain adapter, ploy-N, and reads with low quality (score 5) were removed. Clean reads were then used for subsequent analyses. Transcript expression levels were estimated using fragments per kilobase per million reads (FPKM) values and quantified by RSEM software. EdgeR software was used to identify differential expressed genes ^26, 27^ . The package employed robust statistical models even for small numbers of replicates.

**Gene Ontology and Kyoto Encyclopedia of genes and genomes enrichment analysis***.* For functional enrichment analysis, all DEGs were mapped to terms in the GO databases, and then significantly enriched GO terms were searched for among the DEGs using p < .05 as the threshold. GO term analysis was classified into three subgroups, namely biological process (BP), cellular component (CC) and molecular function (MF). All DEGs were mapped to the KEGG database, and searched for significantly enriched KEGG pathways at p < .05 level.

**Validation of gene expression by quantitative real time polymerase chain reaction***.* To validate the reliability of the DEG results, the expression levels of 7 selected transcripts were determined by RT- qPCR with the housekeeping gene GAPDH and RNA 18S as an endogenous reference. The complete list of primers for all genes and GAPDH/RNA 18S is presented in **Table 1**. Total RNA was extracted for cDNA preparation as described above. Total RNA (1 mg) from MSC samples was reverse transcribed into single stranded cDNA by Revert Aid first strand cDNA Synthesis Kit (Thermo Fisher, Waltham, MA, USA). The incorporation of the SYBR Green dye (Life, Foster City, CA, USA) into the PCR products was monitored in real time with the ABI Prism 7500 PCR system. Reactions were carried out with the following amplification conditions: initial denaturation at 50 ºC for two minutes, 95 ºC for 10 min, followed by 40 cycles of reaction at 95 ºC for 15 s and 60 ºC for 60 s. To confirm the specificity of the amplification reaction, a melting curve was performed after the last amplification cycle. To get reliable calculation of statistical significance, all PCR reactions were performed in triplicate for per differentially expressed gene. The relative quantification of the gene expression was determined normalizing the data of the gene to GAPDH and RNA 18S housekeeping gene and using the 2^−ΔΔCT^ method.

**Statistical analysis.** The edge R package was used, which modelled the gene level read count data assuming a negative binomial distribution and employed the over dispersed Poisson model and an empirical Bayes procedure to moderate the degree of over dispersion across genes, to identify significant DEGs. DEGs were evaluated by meeting the two criteria: (i) more than twofold change in expression; (ii) adjusted p < .05. Log2 scale of fold change was used for convenience. log2(fold change)j > 1 means at least twofold change. Raw p values were adjusted for multiple testing using Bonferroni correction. padj < .05 was considered significant.

**2 Supplementary Figures and Tables**

**Table 1.** Median percentage of survival of MSCs at increasing concentrations of mitomycin C, in the two main study groups (FA-MSCs, HD-MSCs). NS: non significant.

| **Mitomycin C (nM)** | **FA-MSC**  Median (IQR) | **HD-MSC**  Median (IQR) | **p value** |
| --- | --- | --- | --- |
| 0nM | 100.00 (99.99-100.00) | 100.00 (99.99-100.00) | NS |
| 3nM | 78.80 (64.43-89.93) | 96.07 (95.01-97.48) | <0.05 |
| 10nM | 65.72 (44.97-73.43) | 93.18 (86.17-95.05) | <0.05 |
| 33nM | 53.72 (32.11-64.64) | 83.01 (69.08-92.74) | <0.05 |
| 100nM | 49.33 (32.79-54.70) | 60.94 (53.87-82.93) | <0.05 |
| 333nM | 43.21 (33.23-58.20) | 55.93 (44.44-65.64) | NS |

**Table 2.** Median percentage of survival of MSCs with increasing concentrations of mitomycin C, according to study subgroups (B-MSC, GT-MSC, HSCT-MSC). NS: non significant.

| **Mitomycin C (nM)** | **B-MSC**  Median (IQR) | **GT-MSC**  Median (IQR) | **HSCT-MSC**  Median (IQR) | **p value** |
| --- | --- | --- | --- | --- |
| 0nM | 100.00 (99.99-100.00) | 100.00 (100.00-100.00) | 99.99 (99.99-100.00) | NS |
| 3nM | 79.95 (76.28-85.38) | 89.93 (64.43-100.00) | 64.62 (53.81-90.86) | NS |
| 10nM | 65.50 (59.32-68.84) | 73.43 (44.98-100.00) | 47.29 (28.76-88.28) | NS |
| 33nM | 51.19 (47.56-61.91) | 58.95 (32.12-89.01) | 41.61 (22.00-80.37) | NS |
| 100nM | 51.49 (44.89-53.97) | 49.33 (32.80-85.75) | 33.98 (18.90-72.65) | NS |
| 333nM | 46.41 (40.33-56.06) | 48.80 (33.24-84.63) | 29.53 (20.46-55.67) | NS |

**Table 3.** Cumulative population doublings (PDs) from passage (P)1 to P5 of MSCs isolated from Fanconi Anemia (FA) patients prior and after gene therapy.

| **Paired samples**  **(GT)** | **Pre-GT**  Median (IQR) | **Post-GT**  Median (IQR) |
| --- | --- | --- |
| P1-P5 | 594.86  (178.99-822.98) | 19,710.92  (442.44-31,891.91) |

**Table 4.** Cumulative population doublings (PDs) from passage (P)6 to P19 of MSCs isolated from healthy donors (HDs) and from Fanconi Anemia (FA) patients. NS: non significant.

|  | **HD-MSC**  Median (IQR) | **FA-MSC**  Median (IQR) | **P value** |
| --- | --- | --- | --- |
| P6-P19 | 870.48  (40.16-12,873.98) | 861.60  (2.00-41,082.16) | NS |

**Table 5.** Fibroblast-colony forming unit (CFU-F) ability of GT-MSCs obtained before and after treatment with gene therapy (GT).

| **CFU-F** | **Prior-GT**  Median (range) | **After-GT**  Median (range) |
| --- | --- | --- |
| CFU-F | 10.00 (6.50-12.75) | 2.50 (2.00-3.00) |

**Table 6.** Ability to support long-term hematopoiesis of the different FA-MSC subgroups (gene-therapy, GT-MSC or hematopoietic stem cell transplantation, HSCT-MSC) as compared to HD-MSCs. NS: non significant.

| **CFC** | **HD-MSC**  Median (IQR) | **B-MSC** Median (IQR) | **GT-MSC** Median (IQR) | **HSCT- MSC** Median (IQR) | **P value** |
| --- | --- | --- | --- | --- | --- |
| CFC-F/10.000 | 5.96  (1.98-25.55) | 6.65  (3.81-21.93) | 4.90  (2.73-11.92) | 12.07  (8.68-24.98) | 0.016 |

**Table 7.** Ability to support long-term hematopoiesis of GT-MSCs obtained before and after treatment.

| **CFC** | **Prior-GT**  Median (IQR) | **After-GT**  Median (IQR) |
| --- | --- | --- |
| FA-MSC | 6.35 (3.81-20.84) | 6.88 (2.81-13.67) |

**Figure 1.** Senescence assessed by b-galactosidase staining. FA-MSC, ID 15.

**
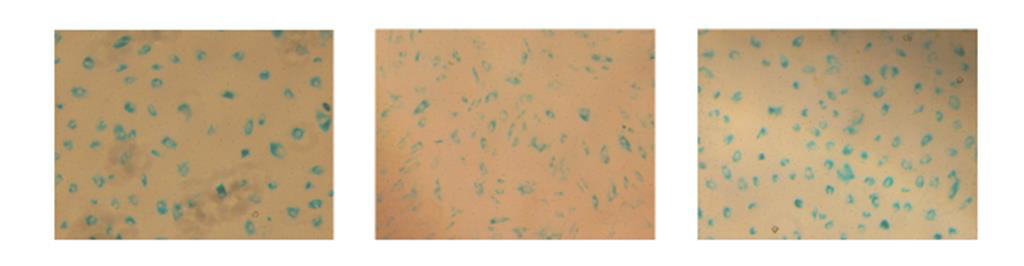
**
